# A migrant study of pubertal timing and tempo in British-Bangladeshi girls at varying risk for breast cancer

**DOI:** 10.1186/s13058-014-0469-8

**Published:** 2014-11-15

**Authors:** Lauren C Houghton, Gillian D Cooper, Gillian R Bentley, Mark Booth, Osul A Chowdhury, Rebecca Troisi, Regina G Ziegler, Robert N Hoover, Hormuzd A Katki

**Affiliations:** 1 Division of Cancer Epidemiology and Genetics, National Cancer Institute, National Institutes of Health, Bethesda, 20892 MD USA; 2 Department of Anthropology and Wolfson Research Institute for Health and Wellbeing, Durham University, Durham, UK; 3 School for Medicine, Pharmacy and Health, Durham University, Durham, UK; 4 Sylhet MAG Osmani Medical College, Sylhet, Bangladesh

## Abstract

**Introduction:**

Earlier menarche is related to subsequent breast cancer risk, yet international differences in the age and tempo of other pubertal milestones and their relationships with body mass index (BMI) are not firmly established in populations at differing risk for breast cancer. We compared age and tempo of adrenarche, thelarche, pubarche, and menarche in a migrant study of Bangladeshi girls to the United Kingdom (UK) and assessed whether differences by migration were explained by differences in BMI.

**Methods:**

Included were groups of Bangladeshi (*n* =168), British-Bangladeshi (*n* =174) and white British (*n* =54) girls, aged 5 to 16 years. Interviewer-administered questionnaires obtained pubertal staging; height and weight were measured. Salivary dehydroepiandrosterone-sulfate concentrations >400 pg/ml defined adrenarche. Median ages of pubertal milestones and hazard ratios (HR) with 95% confidence intervals (CI) were estimated from Weibull survival models.

**Results:**

In all three groups, adrenarche occurred earliest, followed by thelarche, pubarche, and finally menarche. Neither median age at adrenarche (Bangladeshi = 7.2, British-Bangladeshi = 7.4, white British = 7.1; *P*-trend = 0.70) nor at menarche (Bangladeshi = 12.5, British-Bangladeshi = 12.1, white British = 12.6; *P*-trend = 0.70) differed across groups. In contrast, median age at thelarche (Bangladeshi = 10.7, British-Bangladeshi = 9.6, white British = 8.7; *P*-trend <0.01) occurred earlier among girls living in the UK. Compared with Bangladeshi girls, HRs (95% CI) for earlier thelarche were 1.6 (1.1 to 2.4) for British-Bangladeshi girls and 2.6 (1.5 to 4.4) for white British girls (*P*-trend <0.01), but were attenuated after adjustment for BMI (British-Bangladeshi = 1.1 (0.7 to 1.8), white British = 1.7(1.0 to 3.1); *P*-trend =0.20).

**Conclusions:**

Thelarche occurred earlier, but puberty progressed slower with increasing exposure to the UK environment; differences were partially explained by greater BMI. The growth environment might account for much of the ethnic differences in pubertal development observed across and within countries.

**Electronic supplementary material:**

The online version of this article (doi:10.1186/s13058-014-0469-8) contains supplementary material, which is available to authorized users.

## Introduction

**Figure 1:**
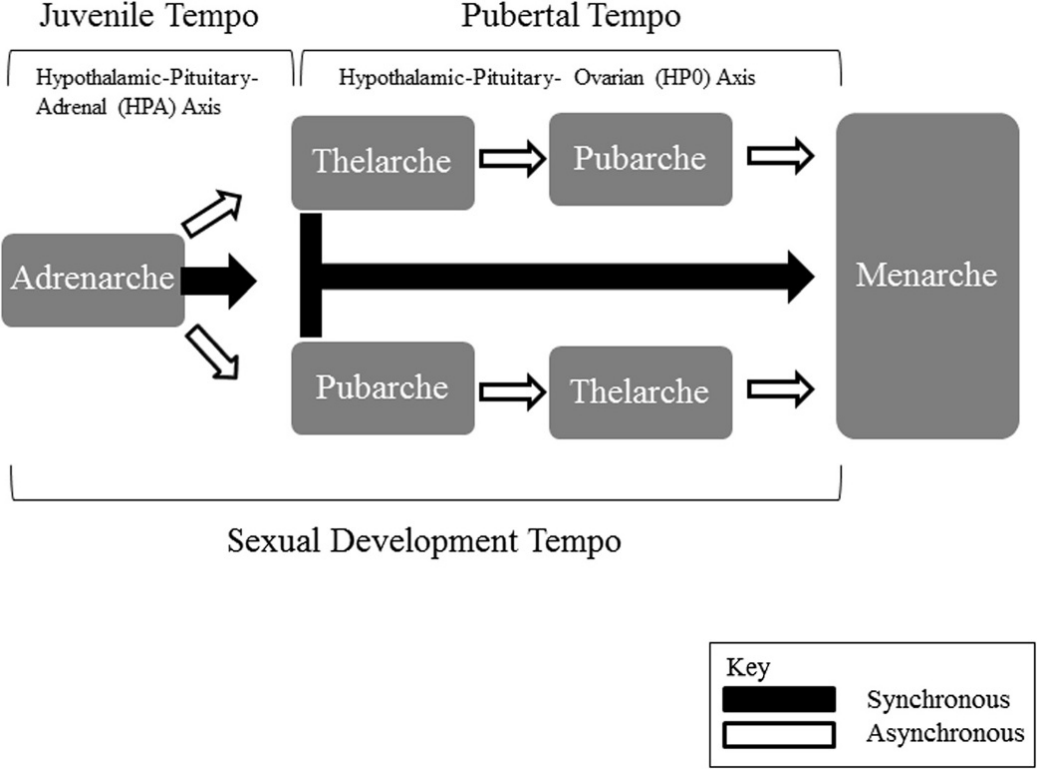
**Theoretical model of sexual development.** The model above distinguishes adrenarche from pubarche and marks adrenarche as the first change to occur during juvenile development. A child could then progress from adrenarche to menarche, synchronously or asynchronously, through either the pubarche or thelarche pathway. The timing of each milestone is equivalent to the age of onset, whereas, the tempo is determined by the interval of time between consecutive milestones.

Menarche is one of the longest known and well-established early-life risk factors for breast cancer [1]. However, menarche is the final milestone of a complex, integrated series of biological events - the pubertal transition. This includes adrenarche (onset of substantial adrenal androgen production), thelarche (onset of breast development), pubarche (onset of pubic hair growth), and finally menarche. The timing (or age) at each of these milestones, and the intervals between them (the tempo of the transition) (Figure 1), are also relevant to breast cancer risk. In particular, an earlier age at thelarche and a longer interval between thelarche and menarche have recently been associated with an increased risk in breast cancer [2]. It may be that molecular changes due to hormonal or environmental factors during the development of the mammary gland (thelarche) itself may be the operative mechanism beneath the menarche/ breast cancer association.

There have been numerous studies of international differences in age at menarche [3], but no study has compared the age and tempo of puberty (including adrenarche) among populations at varying risks of breast cancer. Breast cancer incidence in Bangladesh is five times lower than in the UK [4]. However, South Asians in Britain have a rate double that in South Asia [5]. We designed the ABBY Project (Adolescence among Bangladeshi and British Youth) [6], a migrant study of Bangladeshi girls in the UK, to test whether the growth environment (Bangladesh versus UK) and/or ethnicity (Bangladeshi versus white) were associated with the age and tempo of pubertal milestones. The environment in the UK differs greatly from that in Bangladesh, specifically with regard to nutrition, obesity, and exposure to infectious diseases and/or endocrine disrupting chemicals [7], all of which are likely to affect the age and tempo of pubertal milestones either independently or in concert with each other.

## Methods

### Participants

Healthy girls aged 5 to 16 years were recruited from two settings: 1) 10 schools in London, England from September 2009 until December 2010, and 2) 7 schools in Sylhet Town, Bangladesh from January 2011 until April 2011. Selecting schools as the recruitment sites provided a central location for data collection. London schools were chosen in an area in which there would be a high percentage of immigrants from Sylhet, Bangladesh. White British girls were recruited from the same schools as British-Bangladeshi girls. In Sylhet Town, we identified semi-private/semi-government schools, which were likely to have pupils from families who had relatives living in the UK and were of the same socioeconomic status as those families that had emigrated.

The ABBY Project received ethical permission from the Department of Anthropology, Durham University Ethics Committee and the Sylhet MAG Osmani Medical College. The Office of Human Research Subjects at the National Cancer Institute issued an Institutional Review Board exemption based on the existing approvals. Parents provided written informed consent granting their daughters, who provided written informed assent, permission to partake in the interview.

### Residency scale

Participants (*n* = 411) were classified based on birthplace, parents’ birthplace and self-reported ethnicity. The three study groups were: Bangladeshi (B) (n = 168), British-Bangladeshi (BB) (n = 174) and white British (WB) (n = 54). An ordinal variable called the residency scale was created based on the number of individual/ancestral generations resided in the UK. Bangladeshi girls (residency scale = 0) were born and resided in Bangladesh with no ancestral generations who lived in the UK; second generation British-Bangladeshi girls (residency scale = 2) were born and resided in the UK with one ancestral generation who lived in the UK, and white British girls (residency scale = 3) were born and resided in the UK with at least two ancestral generations who lived in the UK. This scale was created to test for a trend according to increasing exposure to the UK growth environment, a surrogate measure for a range of environmental exposures, including an obeseogenic diet and/or endocrine disruptors.

### Questionnaire and data collection

Study participants were interviewed in person using a standardised questionnaire. The questionnaire was used to collect information on the girl’s date of birth, family history of migration, and ethnicity. The girls reported whether they had reached menarche at the time of interview and if so, the age (years and month) when it occurred. Thelarche and pubarche were assessed by self-report using a modified version of the pubertal development scale (PDS) and were defined as the PDS equivalent to Tanner Stage 2 [8],[9]. We previously validated the self-reported Tanner staging by comparing urinary oestrogen levels between each stage [6].

Saliva samples were collected at the time of interview (between 09:00 and 16:00 hours) for measurement of dehydroepiandrosterone-sulfate (DHEAS) levels. Samples were collected in 5-ml polystyrene tubes using gum base (Cafosa ©, Barcelona, Spain) as a stimulant. Collection tubes were placed immediately in a cooler with ice until transported to the field laboratory in either Bangladesh or London. The samples were stored in −20°C freezers, and then couriered on ice to the Durham Ecology and Endocrinology Laboratory. A total of 377 saliva samples were analysed by one researcher (GC) using a commercially available salivary DHEAS enzyme-linked immunosorbent assay purchased from Salimetrics (State College, PA, USA). The reproducibility of the assay was assessed by two pooled quality-control samples in each batch. The total (within- and between-batch) coefficients of variation were <20%. The lower and upper limits of detection were 43 pg/ml and 16,000 pg/ml, respectively. Adrenarche is clinically defined when DHEAS levels exceed 40 to 50 μg/dl in serum [10],[11] and the corresponding level in saliva was converted using the 0.1% conversion factor [12]. The threshold at which girls were considered to have reached adrenarche was defined as salivary DHEAS levels >400 pg/ml.

Height and weight were measured by two researchers in the UK using standardised techniques [13], and one of the researchers (LCH) took all measurements in Bangladesh. Body mass index (BMI) was calculated (weight in kg/height in m^2^).

### Statistical analyses

Multiple linear regression models were used to evaluate differences in anthropometric measurements among populations. Height, weight, and BMI were compared among study groups stratified into two age groups according to the median age of the sample (<9.5 years and 9.5+ years). We calculated age-standardised BMI *z*-scores and categorised them into quartiles. BMI *z*-scores were compared to reference UK growth-curves to determine nutritional status, reported as the percentage of girls who were clinically underweight, normal weight, overweight, or obese [14],[15].

The key feature of the pubertal data is that the precise age at onset of each pubertal milestone was not known for individual girls. That is, girls were right-censored if they had not reached the milestone and girls who reached the milestone at some unknown age in the past were left-censored. This is an example of current-status data [16]. Because standard Kaplan-Meier and Cox models have difficultly accounting for both left- and right-censoring, estimates for median ages at adrenarche, thelarche, pubarche, and menarche were modelled using flexible Weibull regression models for survival analysis using STATA Version 11.2 (STATA Corporation, College Station, Texas, USA). This method is analogous to the status quo method [17]. Goodness-of-fit was assessed by graphical inspection of the Weibull survival curves with non-parametric survival estimates akin to Kaplan-Meier that account for left/right-censoring (data not shown). Recalled age at menarche was also modelled using standard survival Cox models and there were no differences in median ages at menarche between the Cox and Weibull models. Therefore, to be consistent with the other milestones, the Weibull model was also used to estimate median age at menarche.

From the Weibull models, we derived two types of estimates to assess pubertal age: median age and hazard ratio (HR). The median age at each pubertal milestone is the corresponding age at which 50% of the girls in each group were predicted to have reached that milestone. Tests for trend were performed according to the residency scale. The HR reflects the risk of reaching a milestone at a given age, meaning that a higher ratio indicates an earlier age at onset for the milestone. HRs for the association between ages of adrenarche, thelarche, pubarche, and menarche with residency scale were estimated both unadjusted and adjusted for BMI *z*-scores. To determine if the ages of adrenarche, thelarche, pubarche, and menarche were associated with BMI among all girls, HRs for the association between the onset of each pubertal milestone and BMI *z*-score quartiles were also estimated. To account for possible diurnal variation in DHEAS production, we also adjusted for the time of saliva collection, but this did not change the results (data not shown).

The order of puberty was assessed by comparing the median age of each milestone within each population. Juvenile, pubertal, and sexual development tempo (See Figure 1 for definitions), and the interval between thelarche and pubarche, were calculated for each group and compared by the chi-square test. Statistical significance for all analyses was defined as *P* <0.05.

## Results

### Anthropometrics

Height, weight, and BMI all increased with increasing residency scale, except for height in girls under age 9.5 years (Table 1). Higher percentages of both British-Bangladeshi and white British girls were classified as overweight or obese compared with Bangladeshi girls (Table 1).

**Table 1:** **Anthropometric and nutritional characteristics of Bangladeshi, British-Bangladeshi and white British girls, stratified by age**

Variables	Bangladeshi	British-Bangladeshi	White British	*P* -value
	Mean (SD)	Mean (SD)	Mean (SD)	
Number				
All ages	168	174	54	
<9.5 yr	92	80	26	
≥9.5 yr	76	94	28	
Height, cm				
<9.5 yr	116.4^†^ (9.8)	124.8 (9.9)	126.5 (7.9)	<0.0001
≥9.5 yr	144.0 (11.3)	147.9 (9.8)	144.9 (11.8)	0.07
Weight, kg				
<9.5 yr	37.8^†^ (10.6)	45.9 (12.3)	44.9 (13.8)	<0.01
≥9.5 yr	20.5^†^ (5.8)	28.0 (8.3)	29.6 (6.4)	<0.0001
Body mass index, kg/m^2^				
<9.5 yr	15.0^†^ (3.0)	17.6 (3.1)	18.3 (2.5)	<0.0001
≥9.5 yr	17.9^†^ (3.1)	20.5 (3.6)	20.9 (4.1)	<0.01
Waist circumference, cm				
<9.5 yr	48.9^†^ (6.4)	58.7 (7.4)	61.0 (5.4)	<0.0001
≥9.5 yr	58.3^†^ (6.9)	67.8 (9.3)	68.1 (9.1)	<0.01
Body mass index *z*-score				
<9.5 yr	−0.87^†^ (1.4)	0.53 (1.3)	0.95 (1.1)	<0.0001
≥9.5 yr	−0.41^†^ (1.1)	0.69 (1.1)	0.99 (1.1)	<0.01
Nutritional status^a^				
<9.5 yr				
Underweight	47%	8%	4%	
Normal weight	46%	62%	39%	
Overweight	4%	21%	48%	
Obese	3%	10%	9%	
≥9.5 yr				
Underweight	24%	6%	0%	
Normal weight	67%	60%	60%	
Overweight	7%	27%	19%	

Girls were separated into groups determined by the median age (9.5 years) of the sample. ^a^Nutritional status was derived from body mass index *z*-scores and related to UK clinical references. ^†^Pair-wise comparisons showed that Bangladeshis were significantly different from each group living in the UK, but no other groups were significantly different from each other.

### Age at onset of pubertal milestones

**Figure 2:**
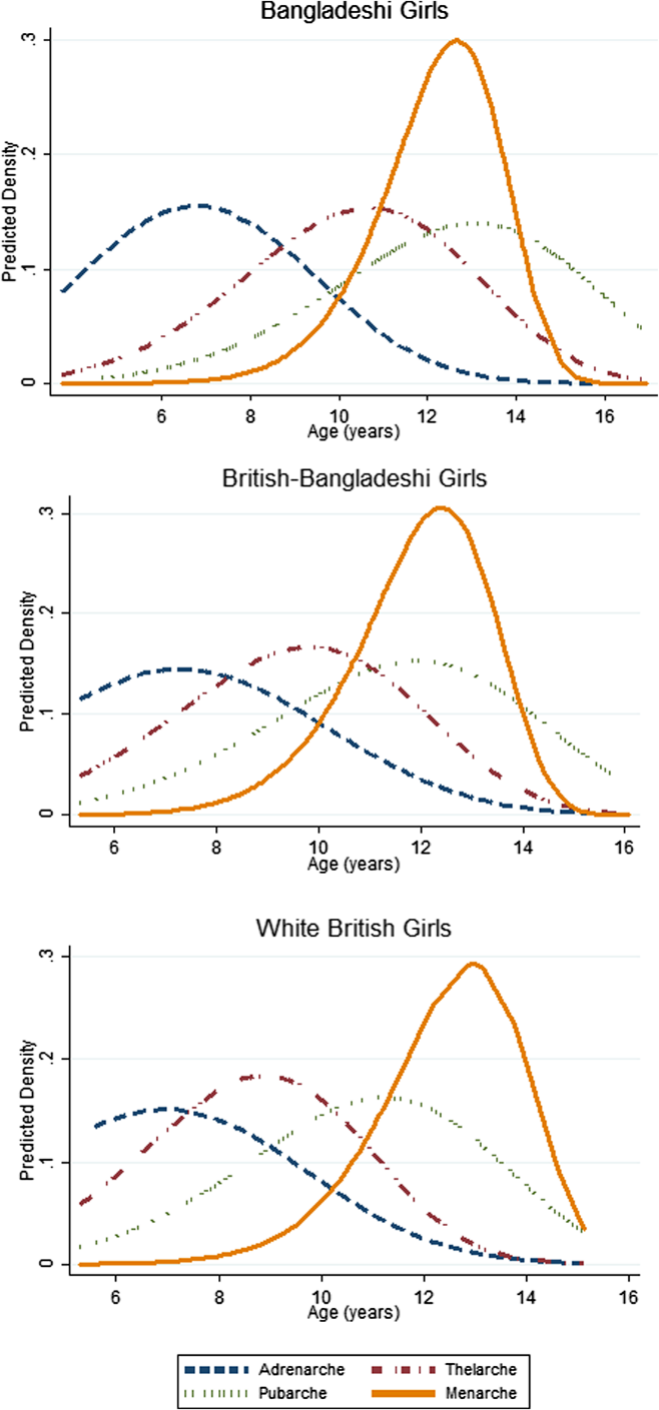
**Comparison of the distribution of ages at adrenarche, thelarche, pubarche, and menarche in Bangladeshi, British-Bangladeshi, and white British girls.** The median age (years) at adrenarche for each population was: (B) = 7.2, British-Bangladeshi (BB) = 7.4, white British (WB) = 7.1; p-trend = 0.70. The median age (years) at thelarche for each population was: B = 10.7, BB = 9.6, WB = 8.7; *P*-trend <0.01. The median age (years) at pubarche for each population was: B = 12.5, BB = 11.6, WB = 10.9; *P*-trend <0.001. The median age (years) at menarche for each population was: B = 12.5, BB = 12.1, WB = 12.6; p-trend = 0.70. The order of sexual development was similar across groups and proceeded in the following order: adrenarche (blue), thelarche (red), pubarche (green) and menarche (yellow). The graphs also illustrate that thelarche (red) and pubarche (green) shift to the right across the residency scale, meaning they occur earlier with increasing individual/ancestral generations in the UK.

Median age at thelarche occurred earlier with increasing residency in the UK (Figure 2; Bangladeshi = 10.7, British-Bangladeshi = 9.6, white British = 8.7 years; *P*-trend <0.001), as did pubarche (Bangladeshi = 12.5, British-Bangladeshi = 11.6, white British = 10.9 years; *P*-trend <0.01). In contrast, there was no trend across groups for adrenarche (Figure 2; Bangladeshi = 7.2, British-Bangladeshi = 7.4, white British = 7.1 years; *P*-trend = 0.70) nor menarche (Figure 2; Bangladeshi = 12.5 years, British-Bangladeshi = 12.1, white British = 12.6 years; *P*-trend = 0.70).

Compared with Bangladeshi girls, HRs (95% CI) for reaching thelarche were 1.6 (1.1, 2.4) for British-Bangladeshi girls and 2.6 (1.5, 4.4) for white British girls (Table 2; *P*-trend <0.01). This overall trend of reaching thelarche earlier with increasing residency scale remained after adjustment for BMI, but was no longer statistically significant (Table 2; HR (95% CI): Bangladeshi = 1.0, British-Bangladeshi = 1.1 (0.7, 1.8), white British = 1.7 (1.0, 3.1); *P*-trend = 0.20). In white British girls, BMI explained 83% of the proportion of risk of reaching thelarche earlier than Bangladeshi girls; in British-Bangladeshi girls, BMI explained 56% of the proportion of risk of reaching thelarche earlier than Bangladeshi girls. The HRs (95% CI) for reaching pubarche were 1.5 (1.0, 2.3) for British-Bangladeshi girls and 2.0 (1.1, 3.7) for white British girls compared with Bangladeshi girls (Table 2; *P*-trend <0.001). Reaching pubarche earlier with increasing residency scale remained after adjustment for BMI (Table 2; HR (95%CI): Bangladeshi = 1.0, British-Bangladeshi = 1.6 (1.0, 2.7), white British = 2.2 (1.1, 4.1); *P*-trend = 0.02).

**Table 2:** **Hazard ratio (HR) and 95% CI for onset of adrenarche, thelarche, pubarche and menarche among Bangladeshi, British-Bangladeshi and white British girls**

	Adrenarche			Thelarche			Pubarche			Menarche		
	Number	HR	95% CI	Number	HR	95% CI	Number	HR	95% CI	Number	HR	95% CI
**Unadjusted resident group**												
Bangladeshi	165	1.0		168	1.0		162	1.0		168	1.0	
British-Bangladeshi	162	0.9	0.6, 1.3	159	1.6	1.1, 2.4	165	1.5	1.0, 2.3	174	1.4	0.8, 2.4
White British	50	1.0	0.6, 1.7	48	2.6	1.5, 4.4	49	2.0	1.1, 3.7	54	0.9	0.4, 2.1
*P*-trend		0.71			<0.01			<0.001			0.70	
**Resident group, adjusted for BMI**												
Bangladeshi	158	1.0		163	1.0		158	1.0		165	1.0	
British-Bangladeshi	147	0.7	0.5, 1.1	149	1.1	0.7, 1.8	154	1.6	1.0, 2.7	156	1.0	0.5, 1.9
White British	46	0.9	0.5, 1.5	46	1.7	1.0, 3.1	47	2.2	1.1, 4.1	50	0.6	0.3, 1.6
*P*-trend		0.17			0.20			0.02			0.23	

A higher HR indicates an earlier age at onset compared with the Bangladeshi reference group. BMI, body mass index.

**Figure 3:**
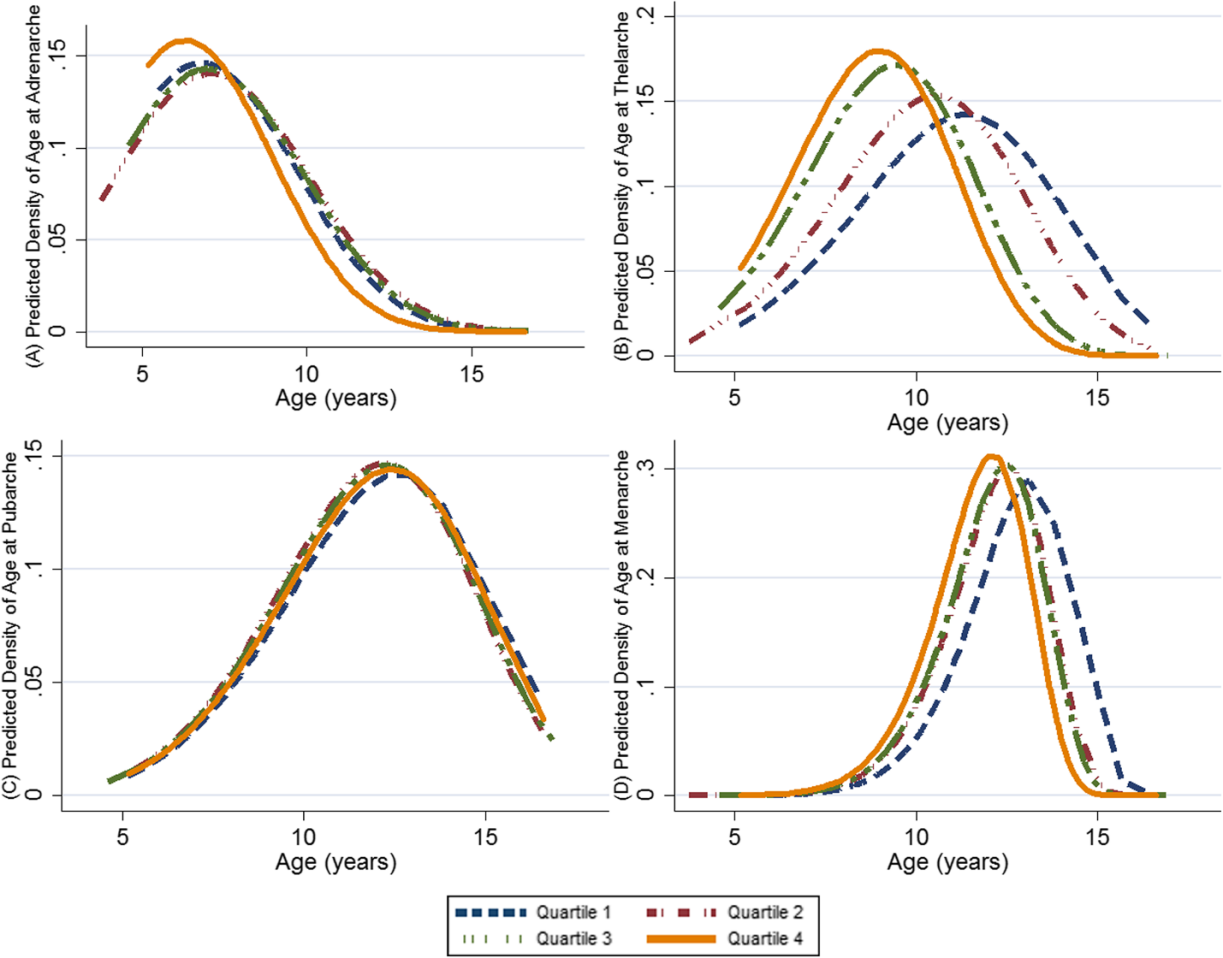
**Density distributions of age at onset of adrenarche, thelarche, pubarche and menarche by body mass index (BMI)** ***z*** **-score quartiles among all girls, aged 5 to 16 years.** The hazard ratios and 95% CI for each pubertal milestone by BMI *z*-score quartile were: **(A)** adrenarche: Q1 = 1.0, Q2 = 1.0 (0.8, 1.2), Q3 = 1.0 (0.8, 1.1), Q4 = 1.1 (1.0, 1.2); *P*-trend = 0.30; **(B)** thelarche: Q1 = 1.0, Q2 = 1.1 (0.9, 1.5), Q3 = 1.3 (1.1, 1.6), Q4 = 1.3 (1.1, 1.5); *P*-trend <0.001; **(C)** pubarche: Q1 = 1.0, Q2 = 1.1 (0.8, 1.5), Q3 = 1.0 (0.9, 1.3), Q4 = 1.0 (0.9, 1.2); *P*-trend = 0.98; **(D)** menarche: Q1 = 1.0, Q2 = 1.2 (0.8, 1.8), Q3 = 1.1 (0.9, 1.5), Q4 = 1.2 (1.0, 1.4); *P*-trend = 0.50.

For all girls, BMI was associated with the age of adrenarche, thelarche and menarche, but not pubarche. The median age at adrenarche was about one year earlier among girls with a BMI *z*-score in the fourth quartile (Q) compared with the first (Figure 3; HR (95% CI): Q1 = 1.0, Q2 = 1.0 (0.8, 1.2), Q3 = 1.0 (0.8, 1.1), Q4 = 1.1 (1.0, 1.2); *P*-trend = 0.30). The median age of thelarche occurred about two years earlier among girls in both the third and fourth quartiles of BMI *z*-scores when compared with girls in the first two quartiles (Figure 3; HR (95% CI): Q =1.0, Q2 = 1.1 (0.9, 1.5), Q3 = 1.3 (1.1, 1.6), Q4 = 1.3 (1.1, 1.5); *P*-trend <0.001). The median age of menarche occurred earlier with increasing BMI *z*-scores, but the trend did not reach statistical significance (Figure 3; HR (95% CI): Q1 = 1.0, Q2 = 1.2 (0.8, 1.8), Q3 = 1.1 (0.9, 1.5), Q4 = 1.2 (1.0, 1.4); *P*-trend = 0.50).

### Order and tempo of sexual maturation

The order of sexual development was similar across Bangladeshi, British-Bangladeshi, and white British girls and proceeded in the following order: adrenarche, thelarche, pubarche, and menarche (Figure 2). Only 10% of girls reported pubarche before adrenarche, 7% of girls reached pubarche before thelarche, and 9% reached menarche before pubarche.

**Figure 4:**
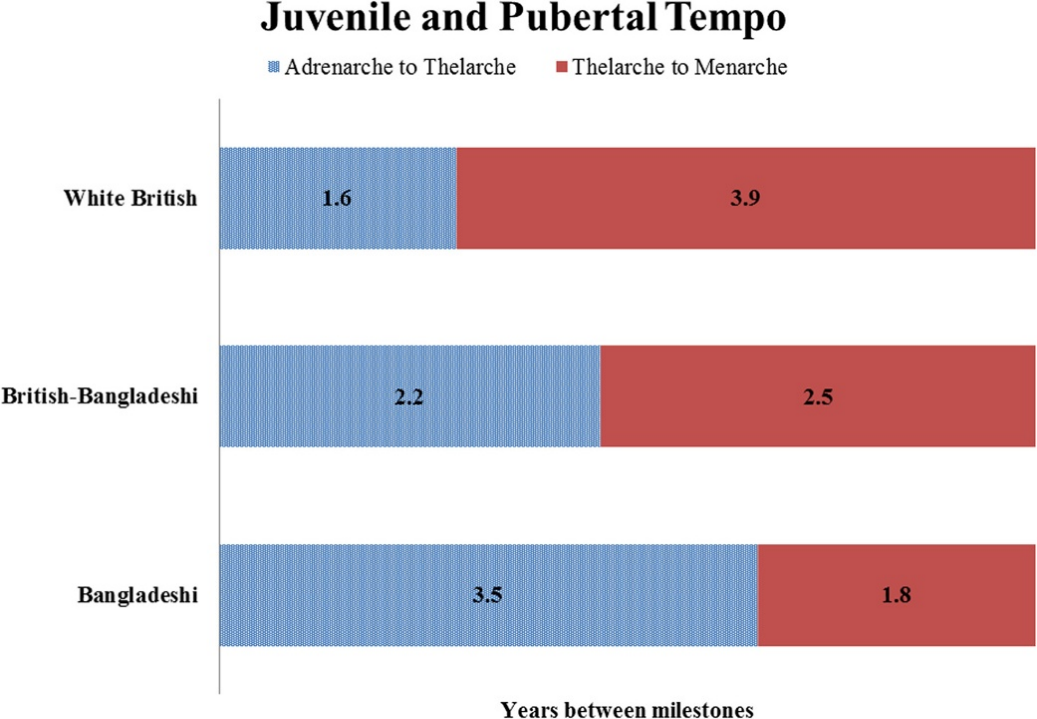
**Tempo of juvenility, puberty, and sexual development among Bangladeshi, British-Bangladeshi and white British girls.** The interval (in years) between adrenarche and thelarche (juvenile tempo) was: Bangladeshi = 3.5, British-Bangladeshi = 2.2, white British = 1.6. The interval between thelarche and menarche (pubertal tempo) was: Bangladeshi = 1.8, British-Bangladeshi = 2.5, white British = 3.9. The differences in tempos among groups were all statistically significant at *P* <0.001.

The tempo of juvenility (the interval between adrenarche and thelarche) was more rapid for girls living in the UK (Figure 4; Bangladeshi = 3.5, British-Bangladeshi = 2.2, white British = 1.6 years; *P* <0.001). However, the tempo of puberty (the interval between thelarche and menarche) was slower for girls living in the UK (Figure 4; Bangladeshi = 1.8, British-Bangladeshi = 2.5, white British = 3.9 years; *P* <0.001). The tempo of sexual development (interval between adrenarche and menarche) was approximately five years among all groups, (Figure 4; Bangladeshi = 5.3, British-Bangladeshi = 4.7, white British = 5.5; *P* <0.001). The interval between thelarche and pubarche was approximately two years in all groups (Figure 4; Bangladeshi = 1.8, British-Bangladeshi = 2, white British = 2.2; *P* <0.001).

## Discussion

We compared the ages of adrenarche, thelarche, pubarche and menarche across groups of girls who differ by growth environment (Bangladesh versus UK) and ethnicity (Bangladeshi versus White). British-Bangladeshi girls begin and progress through puberty earlier and slower than girls living in Bangladesh, but not as early or slowly as white British girls. The differences in pubertal development were partly, but not entirely, explained by higher BMI among white British and British-Bangladeshi girls versus Bangladeshi girls, suggesting that nutritional and other environmental factors associated with the UK growth environment are associated with early, but prolonged, pubertal development.

We are the first to compare adrenarche (hormonally defined) between European and Asian populations, and the ages of adrenarche determined in this study are also consistent with other studies that report adrenarche to occur between ages 6 to 8 years in healthy children [10],[18],[19]. Contrary to expectations, we found no difference in age at menarche among Bangladeshi, British-Bangladeshi or white British girls. The median ages at menarche in our study were consistent with the current ages in national studies [20], which, in Western Europe, have remained between ages 12 to 13 years since the 1960s [21]. Previously, however, there was a secular decline in age at menarche in the early 20^th^ century [3],[22] that is largely attributed to concurrent improvements in general health, nutrition and living conditions [21],[23],[24]. Within Bangladesh, girls with higher socioeconomic status (SES) reach menarche earlier than girls with lower SES [25]. Since the Bangladeshi girls were recruited from middle class families with the means to migrate, their SES may explain the similarities in age at menarche between them and white British girls.

Our findings support secular observations of the age at thelarche continuing to decline [21]. The median ages at thelarche for each study population, even the Bangladeshi girls, were markedly earlier than the mean age at breast stage 2 (11 years) first reported for British girls in the classic Marshall and Tanner study [9]. Our findings are consistent with more recent studies that show breast development occurring between ages 9 to 11 years [26]–[32]. The white British girls in our study, however, reached thelarche particularly early compared with white American girls, but no earlier than black American girls [32],[33]. This discrepancy may be due to differences in study design, but high rates of overweight and obesity in our white population may also explain the particularly early age at thelarche.

The age at pubertal onset is strongly influenced by environmental factors. Increased body fat, possibly mediated by leptin, has been previously linked with an earlier pubertal onset [32],[34]. BMI accounted for about 80% of the variation between Bangladeshi and white British girls, suggesting that differences in dietary composition and quality [35]–[36] between ethnicities may alter the age at pubertal onset. BMI accounted for 55% of the difference in age at thelarche between Bangladeshi and British-Bangladeshi girls suggesting that other factors associated with the UK environment, such as less infectious disease or increased exposure to oestrogenic substances, are also driving the earlier pubertal onset in British-Bangladeshi girls. BMI was not independently associated with pubarche in our data, and variation in pubarche according to residency scale was not explained by BMI, suggesting that other environmental factors are implicated.

This study supports a model whereby most sexual development starts with adrenarche and then proceeds from thelarche to pubarche to menarche. In contrast, other studies have previously distinguished girls that progress through puberty along the thelarche pathway from those that progress through an adrenarche pathway [37]. Our study suggests that adrenarche precedes the physical manifestation of both thelarche and pubarche. The intervals between the age at adrenarche and the age at thelarche or pubarche may reflect underlying differences in the source of androgens and oestrogen production during sexual development.

A slower pubertal tempo, but faster juvenile tempo, was associated with increasing residency in the UK growth environment in our data. An earlier onset of thelarche, but slower progression through puberty, has also been observed over time within the US and Western Europe [23],[38]. Girls growing up in an industrialized growth environment could either be progressing through puberty at a steady, but slower tempo than before or pubertal development in industrialized countries has become more punctuated, meaning that some pubertal milestones appear abruptly and are disjointed from subsequent ones. Punctuated puberty may not reflect an activated hypothalamic-pituitary-ovarian axis, but rather peripheral production of oestrogens from adipose tissue [39] or exposure to endocrine disrupting chemicals [40]. This earlier and prolonged exposure to oestrogenic substances, regardless of source, may be implicated in the increasing rates of breast cancer observed in South Asian populations living in the UK [41],[42]. Alternatively, androgen exposure occurring between the onset of adrenarche and thelarche could be protective for the developing breast. The reduction in juvenile tempo with increasing residency in the UK may explain increasing breast cancer rates in South Asian migrants to the UK [41].

There are limitations that need to be considered when interpreting the findings reported here. First, the comparison of age at menarche across groups was limited by the small sample size in the older white British girls, which resulted in some instability in estimates. However, the consistency in median ages between status quo and recalled methods, suggests that our approach is robust. Secondly, while our cross-sectional findings should be reproduced in longitudinal studies, the migrant study design enabled us to compare migrants of the same ethnicity, in terms of two generations, with girls living in both the “home” and “host” country. Finally, we assessed pubertal staging using self-reported PDS because clinical examinations are not a viable method in a school setting. Although there can be low concordance between findings from physical examination and those from the PDS [43], another study comparing the PDS with both a physical examination and hormone measures found that self-reported breast development and menarche were more reliably related to hormones than the physical examination [44]. Indeed, in our previous study, DHEAS and urinary oestrogen concentrations were higher among girls that reported having secondary sex characteristics compared with girls who did not [6]. Level of BMI can lead to a systematic bias in self-reporting of pubertal staging [44], so it is possible that girls with higher BMI mistook lipomastia for the development of breasts. However, repeating the survival analyses using the progressive stage of breast stage 3 as the end point (as Euling *et al*. (2008) suggest) did not change the overall observed pattern of results. It has also been demonstrated that white adolescents overestimate their pubertal stage more often than non-white adolescents; however within the same Bangladeshi ethnic group, we observed earlier breast development in those living in the UK compared with those living in Bangladesh. Interestingly, there are cultural markers of thelarche within both Bangladeshi populations [6] so it is unlikely that one would overestimate breast development more than the other.

## Conclusion

Our findings suggest that the growth environment might account for the ethnic differences in pubertal age and tempo seen across and within international populations. Bangladeshi girls who grew up in the UK progressed through puberty earlier, but more slowly, than girls who grew up in Sylhet, Bangladesh. BMI explained some, but not all, of these differences in pubertal age. Other environmental factors such as psychosocial stress and endocrine disrupting chemicals may also affect the age and tempo of pubertal milestones. When the tempo between adrenarche, thelarche and menarche are considered together and compared across Bangladeshi, migrant British-Bangladeshi, and white British girls, a clear and provocative pattern emerges: there is a longer period of androgen exposure in populations at low risk, equal periods of androgen and oestrogen exposure in populations at intermediate risk and a prolonged period of oestrogen exposure in populations at high risk. These findings support the inclusion of age of thelarche, a potentially new risk factor, in future breast cancer studies.

## Authors’ original submitted files for images

Below are the links to the authors’ original submitted files for images. Authors’ original file for figure 1 Authors’ original file for figure 2 Authors’ original file for figure 3 Authors’ original file for figure 4

## Abbreviations

ABBY: Adolescence among Bangladeshi and British Youth
BMI: body mass index
DHEAS: dehydroepiandrosterone-sulfate
HR: hazard ratio
PDS: pubertal development scale
