# Supplementary figures and images for: A migrant study of pubertal timing and tempo in British-Bangladeshi girls at varying risk for breast cancer
Source: Breast Cancer Res. 2014 Nov 15;16:469. doi: 10.1186/s13058-014-0469-8 (PMC4303203; doi:10.1186/s13058-014-0469-8)

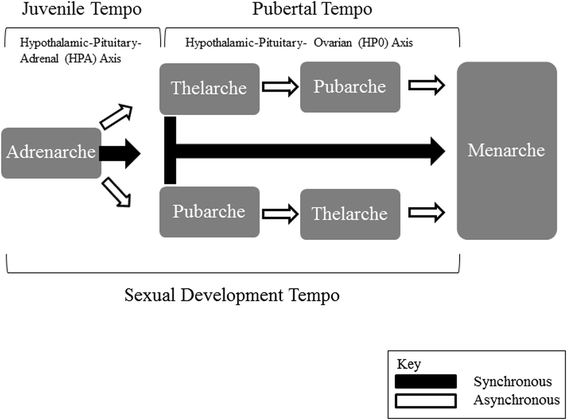

Supplement: Supplementary file 1 — Authors’ original file for figure 1 [file 13058_2014_469_MOESM1_ESM.gif]

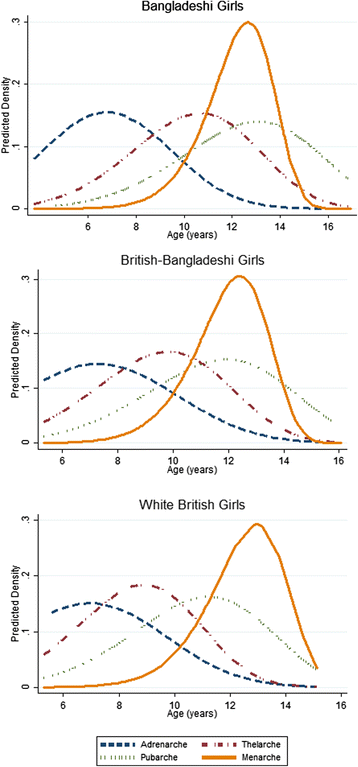

Supplement: Supplementary file 2 — Authors’ original file for figure 2 [file 13058_2014_469_MOESM2_ESM.gif]

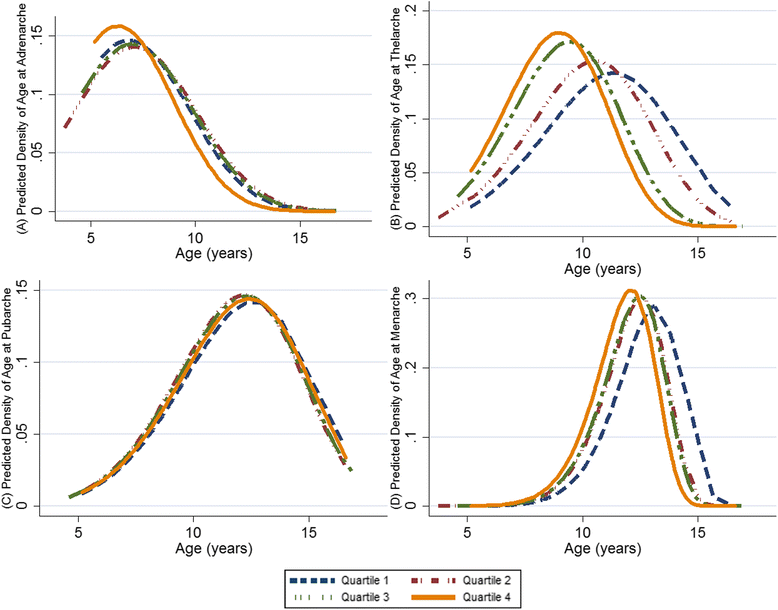

Supplement: Supplementary file 3 — Authors’ original file for figure 3 [file 13058_2014_469_MOESM3_ESM.gif]

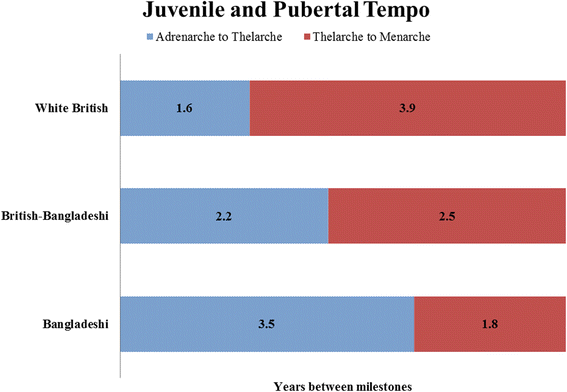

Supplement: Supplementary file 4 — Authors’ original file for figure 4 [file 13058_2014_469_MOESM4_ESM.gif]
